# A Novel Superhard, Wear-Resistant, and Highly Conductive Cu-MoSi_2_ Coating Fabricated by High-Speed Laser Cladding Technique

**DOI:** 10.3390/ma17010020

**Published:** 2023-12-20

**Authors:** Yanmiao Li, Xiaojun Zhao, Pengyuan Zhai, Pengyu Fan, Jiahui Xu, Yuefan Xu, Zengkai Yu, Muyang Li, Yongtong Zhang, Dawei Gao, Sainan Liu, Zhenyang Cai, Lairong Xiao

**Affiliations:** 1 School of Materials Science and Engineering, Central South University, Changsha 410083, China; 213111041@csu.edu.cn (Y.L.); zhaoxj@csu.edu.cn (X.Z.); 8204201302@csu.edu.cn (J.X.); 8204201402@csu.edu.cn (Y.X.); 8204212809@csu.edu.cn (Z.Y.); 0603170518@csu.edu.cn (M.L.); 2 New Technology Promotion Institute of China Ordnance Industries, Beijing 100089, China; 15367833127@163.com (P.Z.); lymsuv@163.com (P.F.); 3 Henan Jianghe Machinery Co., Ltd., Pingdingshan 467337, China; gnjoin@126.com (Y.Z.); ym110611@163.com (D.G.); 4 Center for Mineral Materials, School of Minerals Processing and Bioengineering, Central South University, Changsha 410083, China

**Keywords:** copper-based coating, laser cladding, wear resistance, conductive, first principles

## Abstract

The pursuit of an advanced functional coating that simultaneously combines high hardness, wear resistance, and superior electrical conductivity has remained an elusive goal in the field of copper alloy surface enhancement. Traditional solid solution alloying methods often lead to a significant increase in electron scattering, resulting in a notable reduction in electrical conductivity, making it challenging to achieve a balance between high hardness, wear resistance, and high conductivity. The key lies in identifying a suitable microstructure where dislocation motion is effectively hindered while minimizing the scattering of conductive electrons. In this study, a novel Cu-MoSi_2_ coating was successfully fabricated on a CuCrZr alloy surface using the coaxial powder feeding high-speed laser cladding technique, with the addition of 10–30% MoSi_2_ particles. The coating significantly enhances the hardness and wear resistance of the copper substrate while maintaining favorable electrical conductivity. As the quantity of MoSi_2_ particles increases, the coating’s hardness and wear resistance gradually improve, with minimal variance in conductivity. Among the coatings, the Cu-30%MoSi_2_ coating stands out with the highest hardness (974.5 HV_0.5_) and the lowest wear amount (0.062 mg/km), approximately 15 times the hardness of the copper base material (65 HV_0.5_) and only 0.45% of the wear amount (13.71 mg/km). Additionally, the coating exhibits a resistivity of 0.173 × 10^−6^ Ω·m. The extraordinary hardness and wear resistance of these coatings can be attributed to the dispersion strengthening effect of Mo_x_Si_y_ particles, while the high electrical conductivity is due to the low silicon content dissolved into the copper from the released MoSi_2_ particles, as well as the rapid cooling rates associated with the high-speed laser cladding process.

## 1. Introduction

Copper (Cu) and Cu alloy components are commonly used as electrical conduction and thermal management devices in electricity, pipeline, microelectronic manufacturing, and aerospace industry sectors due to their excellent thermal and electrical conductivity [1,2,3]. Given the growing demand for high-performance copper components across various industries, addressing the inherent challenges of copper’s low hardness and limited wear resistance has become imperative. Manufacturing copper components with exceptional super hardness, impressive wear resistance, and elevated conductivity is now a necessity. Metal matrix composites (MMCs) comprise the properties of metal matrices (ductility and toughness) and reinforcement phases (high strength and stiffness) [4,5,6,7,8], which enables the superior mechanical and functional properties such as excellent wear resistance, controllable coefficient of thermal expansion, and good thermal shock resistance. However, MMCs normally gives rise to notable dislocation pile-ups and severe stress concentration at grain boundaries [9,10]; extensive research has been relying on manipulating the uniform distribution of the reinforcement phase as an innovative approach for designing the microstructural architecture of MMCs to surmount such a dilemma. Some pioneering works have preliminarily confirmed the outstanding advantages of uniformly distributed fine reinforced phases in achieving excellent overall properties in MMCs [11,12]. Recent decades have witnessed remarkable development of MMCs in the field of laser cladding coatings on copper alloys. Gao [13] studied the influence of Cr content on the friction and wear properties of Cu–BCs, and the results showed that coefficient of friction gradually increased with Cr content in the range of 3–5 wt.%. However, due to the high reflectivity of copper surface to laser, poor wettability between materials, complex interaction of parameters, and other factors [14,15,16], it is still challenging to achieve the dispersion distribution of the reinforced phase. In addition, for copper alloys, almost all of these strengthening approaches used to improve mechanical properties are based on the introduction of various kinds of defects that increase the scattering of conducting electrons at these defects, thus increasing the electrical resistivity of copper [17], and there is no optimal solution for the trade-off between the two [18,19,20,21,22]. MoSi_2_, classified as a metal silicide, boasts a range of notable benefits. These include commendable electrical and thermal conductivity, elevated strength and hardness, and impressive resistance against high-temperature corrosion [23,24,25]. Furthermore, owing to its high melting point, MoSi_2_ remains structurally stable without undergoing phase changes within the operational temperature range of copper-based materials [26,27]. This inherent stability positions MoSi_2_ as a promising candidate for serving as a reinforcing agent in the creation of high-strength, wear-resistant, and conductive copper-based composite coatings.

Up to now, there are few reports on the preparation of Cu-MoSi_2_ coatings on copper substrates by laser cladding technology. This work presents a novel coating composed of Cu-MoSi_2_. The microstructure and properties of the Cu-MoSi_2_ coatings are shown, and the good electrical conductivity mechanism of coatings are analyzed by using the first principles. The aim of this work is, therefore, to develop a new coating of copper substrate with high strength, wear resistance, and conductivity; evaluate its conductivity, hardness, and wear resistance; and analyze the underlying mechanisms.

## 2. Materials and Methods

### 2.1. Preparation of Coating

**Figure 1:**
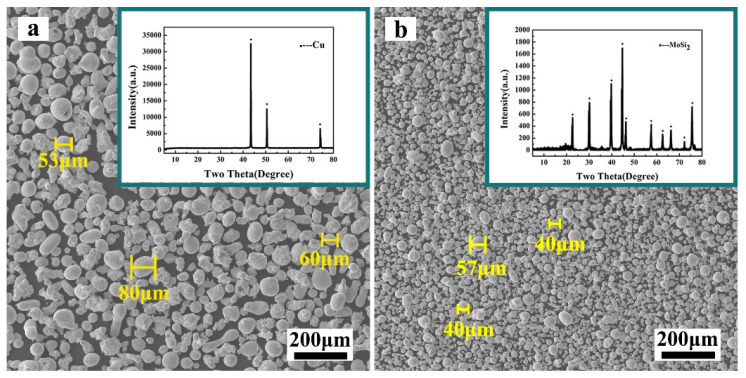
SEM photographs of the powders: (**a**) Cu; (**b**) MoSi_2_.

CuCrZr alloy (Cr: 0.2–1.2%, Zr: 0.03–0.3%, others: max. 0.2%, Cu: residual) is selected as the base material. The copper alloy substrate was cut into 100 mm × 100 mm × 10 mm specimens using the wire cutting machine. The surface of the base material is roughed with 240 mesh sandpaper, then further polished with 600 mesh sandpaper, and then ultrasonic cleaned in 75% alcohol solution for 5 min before drying for use. As shown in Figure 1, spherical copper powder and MoSi_2_ powder(Shanghai Nai’ou Nano Technology Co., LTD., Shanghai, China) were used as cladding raw materials, the purity of raw materials was 3N, and the particle size was 100–300 mesh. Before the cladding experiment, the raw materials should be configured according to the ratio of Cu-*x*MoSi_2_ (*x* = 10, 15, 20, 25, 30, wt.%) and uniformly mixed at the speed of 60 r/min for 9 h (shown in Table 1).

**Figure 2:**
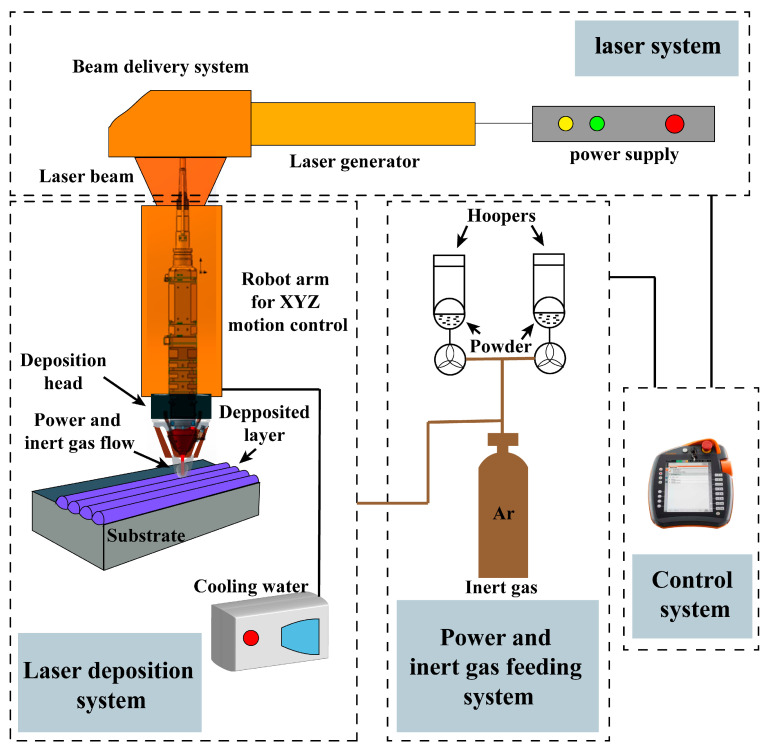
The schematic diagram of laser cladding process.

Laser cladding equipment: ZKZM-6000 W high-speed laser cladding equipment of Shanxi Zhongke Zhongmei Laser Technology Co., Ltd. (Xi’an, China); the laser focusing spot diameter is 5 mm. The laser cladding process diagram is shown in Figure 2. The laser cladding parameters used are as follows: the laser power of the first layer is 4800 W, the scanning speed is 2.8 m/min, the step distance is 1 mm, and the powder feeding amount is 18 g/min; the laser power of the second layer is 3800 W, the scanning speed is 2.8 m/min, the step length is 1 mm, and the powder feeding amount is 18 g/min.

### 2.2. Testing and Characterization

The coating samples of copper alloy substrate obtained by laser cladding were treated with 240 mesh, 400 mesh, 600 mesh, 800 mesh, 1000 mesh, 1200 mesh, and 1500 mesh sandpaper successively. Sigma 2008 A eddy current conductivity meter was used to measure the coating resistivity; 5 positions were randomly selected for measurement and the average value was taken. A 200HBVS-30 Vickers hardness tester was used to determine the Vickers hardness value of the coating; the loading load was 5 N, the load retention time was 15 s; 5 points were randomly hit, and the average value was taken. The wear resistance of the coating was tested by a Zhongke Kaihua GF-I high-speed reciprocating friction and wear testing machine at room temperature (temperature: 20 ± 2 °C, relative humidity: 60 ± 5%). A GCr15 steel ball with a diameter of 4 mm was used as the friction pin, the loading load was 20 N, the rotating speed was 400 r/min, and the friction time was 30 min. A balance (error less than 1 mg) was used to measure the quality of the samples before and after testing, and the average wear rate of the three groups of parallel experiments was taken.

Scanning electron microscopy (SEM, FEI Sirion 200, Hillsboro, OR, USA) and an energy dispersive spectrometer (EDS) were used to characterize the cross-sectional morphology of the coating. A Cu Kα (Rigaku D/max 2500, Tokyo, Japan) X-ray diffractometer (XRD) was used to characterize the coating. Phase analysis was performed. The microstructure of the coating interface was analyzed by field emission transmission electron microscopy (HRTEM) (Titan G2 60-300).

### 2.3. Calculation

Materials Studio2019 and Vesta2020 software were used for crystal structure modeling, CI-NEB of VTST was used for transition state search, and Vasp2022 software was used for initial and final state structure optimization. The state density and differential density distributions of silicon atoms in the copper crystal structure with different solid solubility were obtained by atom diffusion and corresponding energy calculations.

## 3. Results and Discussion

### 3.1. Microstructure Analysis of Coating Section

**Figure 3:**
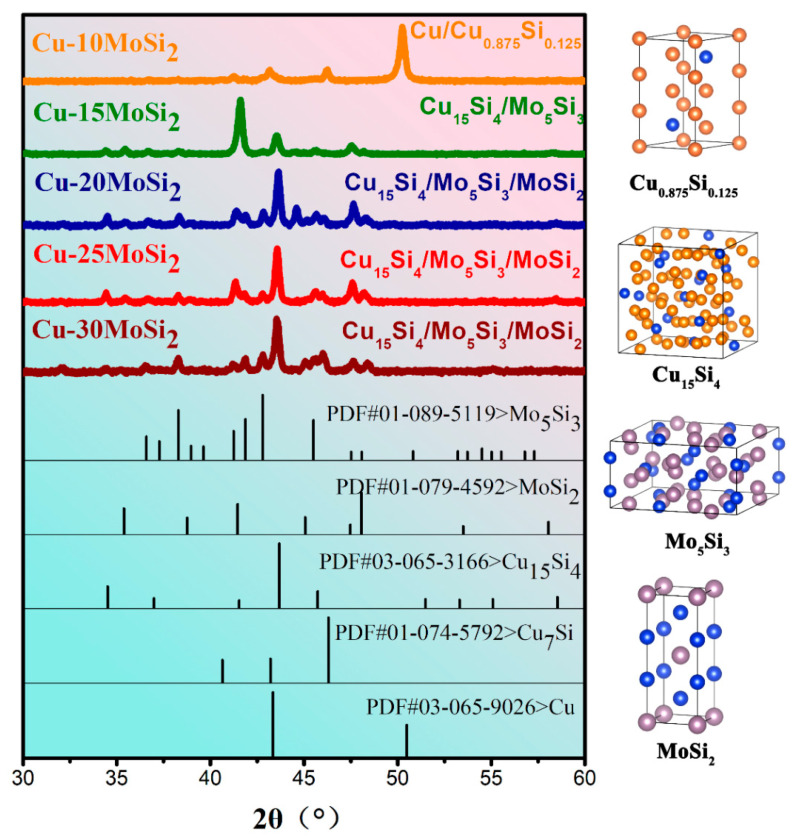
X-ray diffraction results of five composite coatings: Cu-*x*MoSi_2_ (*x* = 10, 15, 20, 25, 30, wt.%) coatings.

The X-ray diffraction results of Cu-*x*MoSi_2_ (*x* = 10, 15, 20, 25, 30, wt.%) coatings are shown in Figure 3. The Cu-10MoSi_2_ coating is mainly composed of Cu and Cu-Si solid solution. With the increase of MoSi_2_ addition, the main phases in Cu-20MoSi_2_, Cu-25MoSi_2_, and Cu-30MoSi_2_ all become MoSi_2_, Mo_5_Si_3_, and Cu_15_Si_4_. XRD results show that both Cu-Si solid solution and Mo_5_Si_3_ phase exist in the coatings, indicating that during the laser cladding process, MoSi_2_ may be decomposed, and the decomposed Si atoms will dissolve with Cu to form Cu-Si solid solution, while the remaining part will be converted into Mo_5_Si_3_ phase with lower Si content [28].

**Figure 4:**
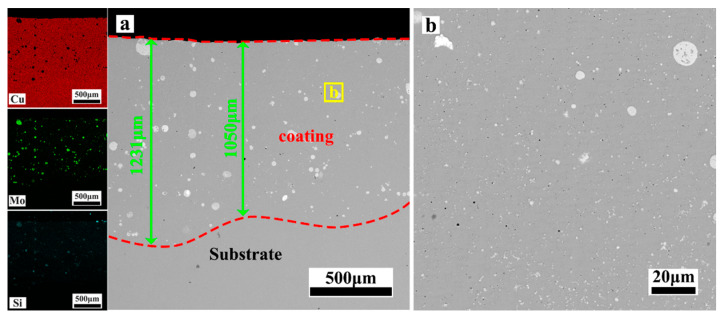
SEM photographs of the cross-sectional microstructure of the coating: (**a**) Cu-10MoSi_2_; (**b**) the enlargement of the areas shown in (**a**).

**Figure 5:**
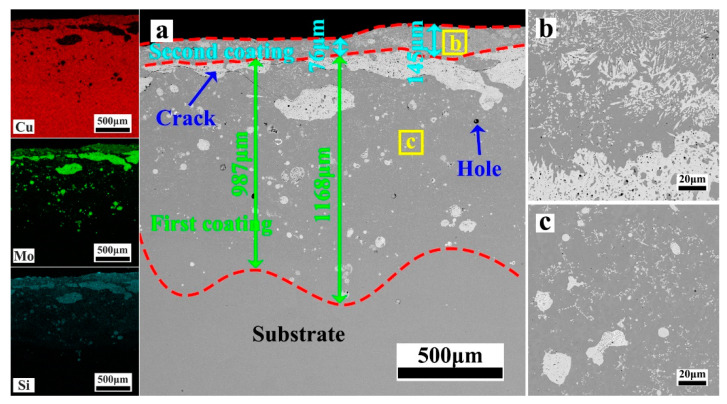
SEM photographs of the cross-sectional microstructure of the coating: (**a**) Cu-15MoSi_2_; (**b**,**c**) are enlargements of the areas shown in (**a**).

**Figure 6:**
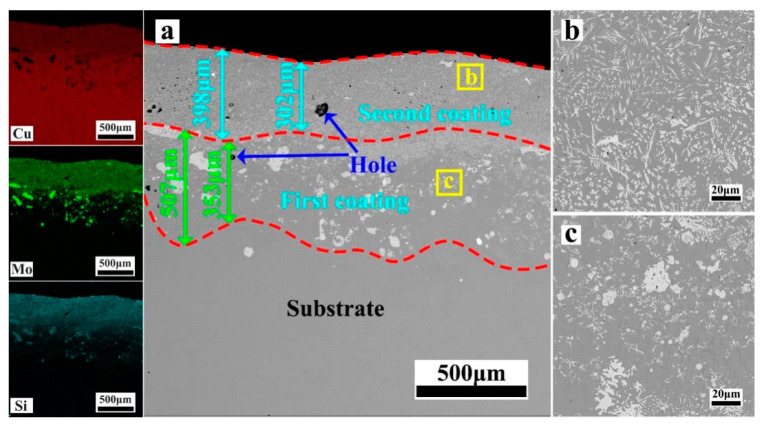
SEM photographs of the cross-sectional microstructure of the coating: (**a**) Cu-20MoSi_2_; (**b**,**c**) are enlargements of the areas shown in (**a**).

**Figure 7:**
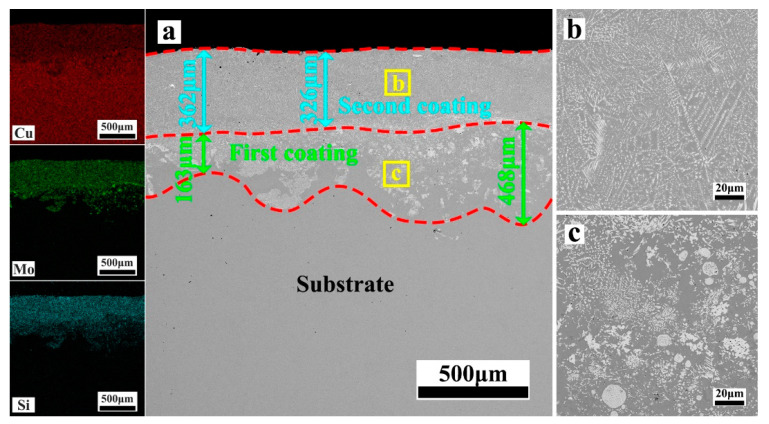
SEM photographs of the cross-sectional microstructure of the coating: (**a**) Cu-25MoSi_2_; (**b**,**c**) are enlargements of the areas shown in (**a**).

**Figure 8:**
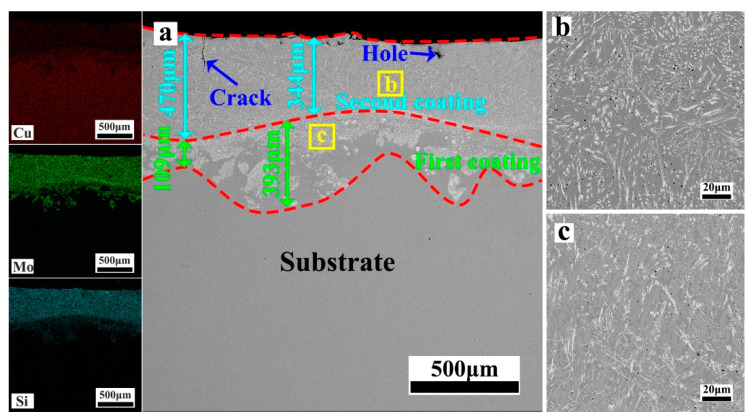
SEM photographs of the cross-sectional microstructure of the coating: (**a**) Cu-30MoSi_2_; (**b**,**c**) are enlargements of the areas shown in (**a**).

Figure 4, Figure 5, Figure 6, Figure 7 and Figure 8 depict the cross-sectional microstructure of Cu-*x*MoSi_2_ (*x* = 10, 15, 20, 25, 30 wt.%) coatings. As observed in Figure 4a, Figure 5a, Figure 6a, Figure 7a and Figure 8a, the coatings exhibit density, and all demonstrate favorable metallurgical bonding with the copper substrate. The coating structure remains similar, primarily comprising two layers. The second layer appears relatively uniform and dense, influenced by the substrate’s laser absorption rate. Upon closer inspection in locally enlarged images, the tissues in the first layer transition from spherical to irregular granular to short needle shapes (Figure 4b, Figure 5b, Figure 6b, Figure 7b and Figure 8b), while those in the second layer evolve from spherical to short needle to long needle shapes (Figure 4b, Figure 5c, Figure 6c, Figure 7c and Figure 8c). Coarse dispersed particles in the coating layer progressively decrease, giving way to fine dispersed needle-like structures. This indicates that an increase in MoSi_2_ content refines the tissue and promotes the dispersion distribution of the enhanced phase. In comparison to the agglomeration of large reinforcement particles, the dispersed fine reinforcement phase significantly reduces stress concentration, contributing to enhanced coating strength. However, it is noteworthy that a higher MoSi_2_ content does not necessarily translate to improved performance. As illustrated in Figure 8, cracks and holes begin to appear in the coating when the MoSi_2_ content reaches 30 wt.%.

**Figure 9:**
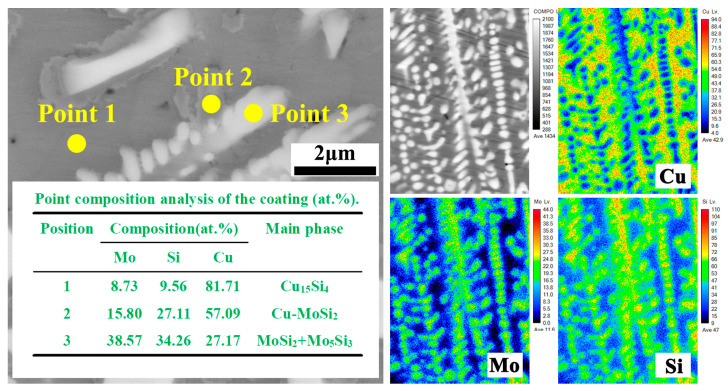
Cross-sectional microstructure and results of element analysis of the Cu-25MoSi_2_ coating.

In order to determine the main component phases, the microstructure in the Cu-25MoSi_2_ coating was observed. The locally enlarged image is shown in Figure 9. Combined with the results of XRD analysis and micro-component analysis, the atom proportion of Cu in the matrix (point 1) is large, and it is inferred that the main component phase is Cu_15_Si_4_. The atomic ratio of Mo and Si in the granular material (point 2) is close to 1:2, and according to the results of XRD analysis, the main component phase is presumed to be Cu-MoSi_2_; the atomic ratio of Mo and Si in the flake material (point 3) is approximately 6:5, and the main component phase is presumed to be MoSi_2_ and Mo_5_Si_3_.

**Figure 10:**
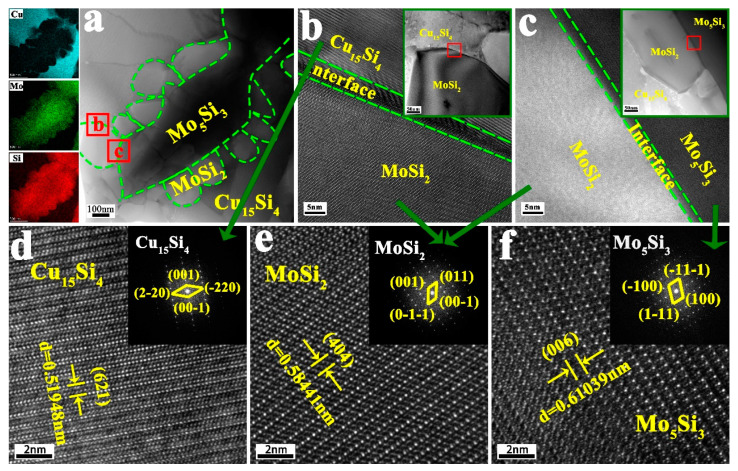
The HRTEM images of the Cu- 25MoSi_2_ coating: (**a**) HRTEM image of Figure 9; (**b**) HRTEM image of area b in (**a**); (**c**) HRTEM image of area c in (**a**); (**d**–**f**) atomic arrangement and diffraction spot analysis of (**b**,**c**).

The HRTEM images of Figure 9 are shown in Figure 10. It can be seen from Figure 10a that there are granular and lamellar phases. Combined with XRD analysis and SEM photo and micro-component analysis results in Figure 9, it can be inferred that the granular phase is mainly MoSi_2_, the lamellar phase is mainly Mo_5_Si_3_, and the matrix is mainly Cu_15_Si_4_. A magnified analysis of the two interfaces, regions b and c in Figure 10a, is shown in Figure 10b,c, respectively. There is a relatively straight interface of about 2 nm between phases. To further determine the composition of the phases, the atomic arrangement results of phases are given in Figure 10d–f. It is clear from the enlarging observation that the interplanar spacings of phases are calculated to be 0.51948 nm, 0.58441 nm, and 0.61039 nm, respectively. These calculations correspond well to the (621) crystal plane of Cu_15_Si_4_, (404) crystal plane of MoSi_2_, and (006) crystal plane of Mo_5_Si_3_. The insets of the electron diffraction pattern in Figure 10d–f further confirm our guesses about the major component phases. 

### 3.2. Performance Analysis

**Figure 11:**
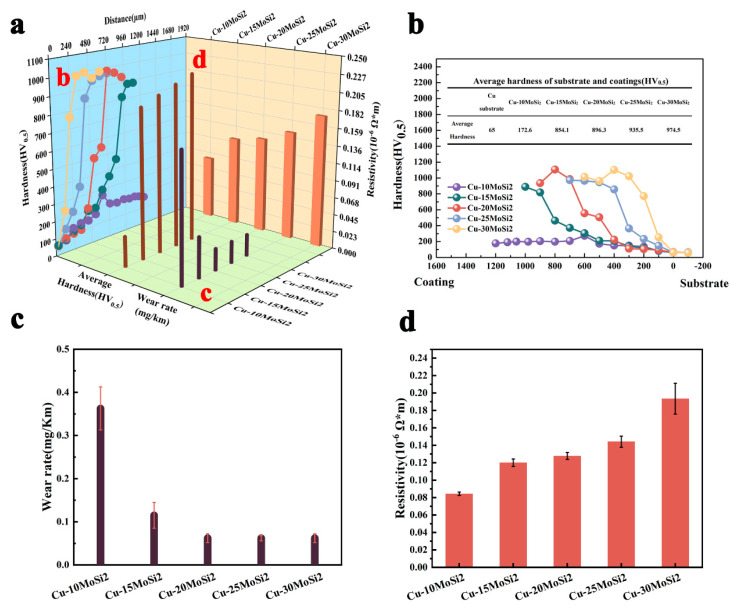
Properties of coatings: (**a**) comprehensive properties; (**b**) hardness; (**c**) wear rate; (**d**) resistivity.

Figure 11 illustrates the properties of hardness, wear resistance, and electrical conductivity in the Cu-MoSi_2_ coating.

#### 3.2.1. Hardness

Figure 11b shows the distribution curve of hardness of coatings from the outside to the inside and its average hardness value. It can be seen that the average hardness of Cu-10MoSi_2_ is only 172.6 HV_0.5_, and the average hardness of Cu-15MoSi_2_ and Cu-20MoSi_2_ is 854.1 HV_0.5_ and 896.3 HV_0.5_, respectively. The hardness of Cu-25MoSi_2_ and Cu-30MoSi_2_ coatings exceeds 900 HV_0.5_, which is 935.5 HV_0.5_ and 974.5 HV_0.5_, respectively, which is about 15 times that of the copper substrate with a hardness of 65 HV_0.5_. This indicates that the addition of MoSi_2_ helps to improve the hardness of the coating. According to the Orowan mechanism [29], combined with the analysis of the coating structure, it can be inferred that the blocking effect of fine and dispersed MoSi_2_-Mo_5_Si_3_ enhanced particles on dislocation leads to the enhancement of coating hardness, which is specifically represented by the intensive enhanced particles-dislocation result in the dislocation proliferation in the coating’s interior, avoiding notable dislocation pile-ups and severe stress concentration at the grain boundaries, thus enhancing the hardness of the coating.

#### 3.2.2. Wear Resistance

Figure 11c compares the wear resistance of the five coatings tested in this research. The wear rate of copper substrate is 13.71 mg/km; the wear rate of Cu-20MoSi_2_ coating, Cu-25MoSi_2_ coating, and Cu-30MoSi_2_ coating is 0.0625 mg/km, 0.0620 mg/km, and 0.0619 mg/km, respectively, which is only 0.45% that of the copper substrate. The wear rate of the coatings is much lower than that of copper substrate. It indicates that Cu-MoSi_2_ coatings can significantly improve the wear resistance of copper substrate. 

**Figure 12:**
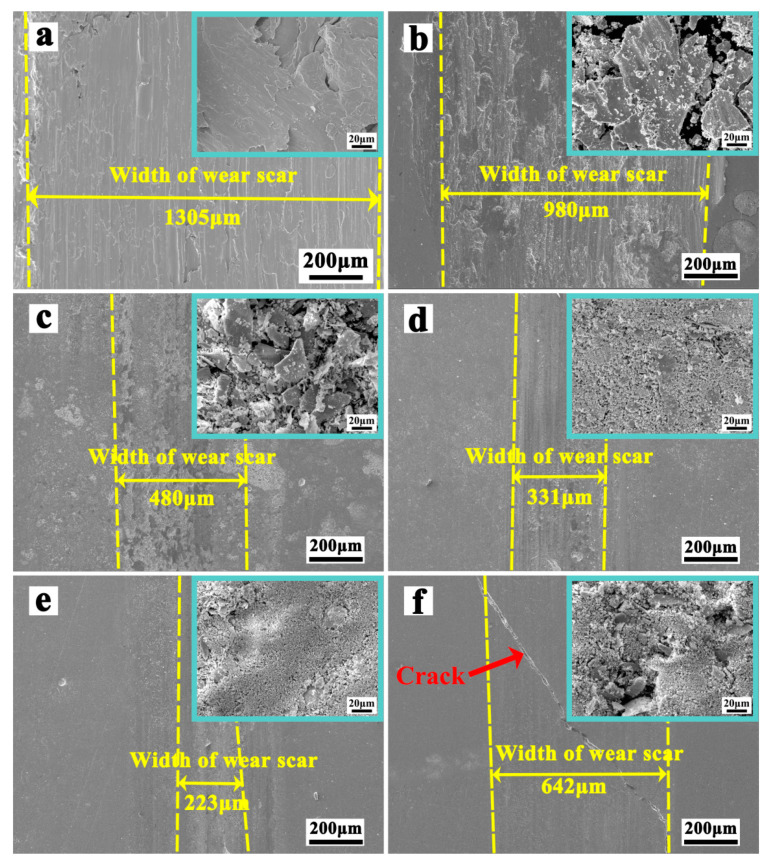
Photographs of substrate and five coatings after the frictional wear test: (**a**) substrate; (**b**) Cu-10MoSi_2_; (**c**) Cu-15MoSi_2_; (**d**) Cu-20MoSi_2_; (**e**) Cu-25MoSi_2_; (**f**) Cu-30MoSi_2_.

Figure 12 shows the SEM photographs of the wear surface of copper substrate and coatings. The wear surface morphology of copper substrate shows noticeable plastic deformation features. The wear debris is flake-like. The wear mechanism of copper substrate is adhesive wear. This can be confirmed by the obvious scratches on the wear surface and the plate-like wear debris [30,31]. The dominance of adhesive wear is due to the significantly higher hardness of the wear ball (GCr15) relative to copper substrate. When the wear ball is against the copper substrate, the copper substrate is plastically deformed and scratched. Further, when the degree of plastic deformation exceeds a certain threshold, the copper substrate begins to form cracks and split from the surface, forming the metallic plate-like wear debris. In sharp contrast to the wear surfaces of copper substrate, only shallow scratches are observed on the wear surfaces of Cu-30MoSi_2_ coatings. The Mo_x_Si_y_ particles should be an important factor in their anti-wear properties. The higher wear resistances of coatings are attributed to their superior hardness [32]. The hardness of Cu-*x*MoSi_2_ (*x* = 15, 20, 25, 30, wt.%) coating is over 850 HV_0.5_; therefore, the coatings are strong enough to resist the wear against the ball, and the coating surface will not be worn off quickly during the wear test. In fact, they are able to withstand being worn for a long time. Compared with the copper substrate, Cu-MoSi_2_ coatings exhibit even higher resistance to adhesive wear.

#### 3.2.3. Conductivity

**Figure 13:**
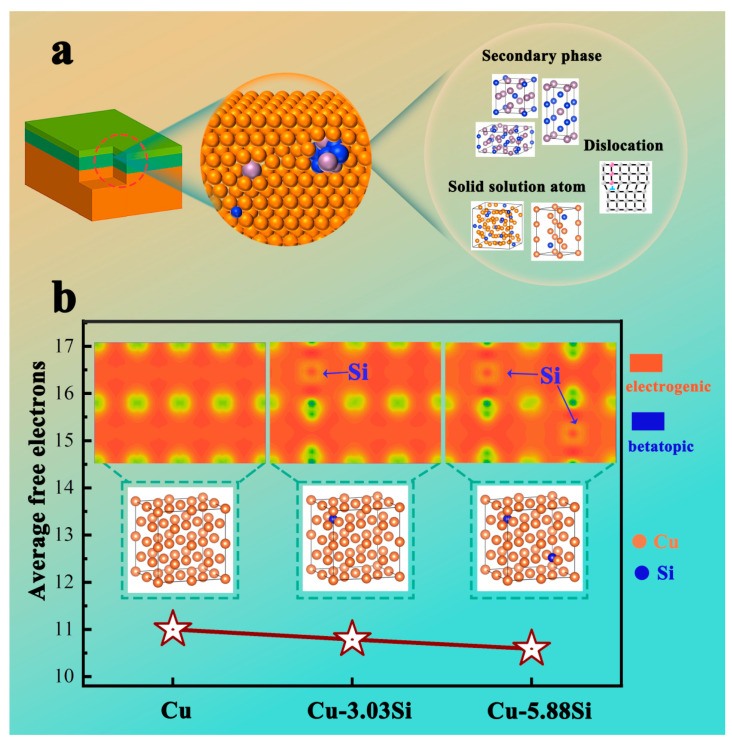
(**a**) Schematic of factors that influence the electrical resistivity of metals; (**b**) the calculated result for Cu-Si.

Figure 11d shows the resistivity of the Cu-*x*MoSi_2_ (*x* = 10, 15, 20, 25, 30, wt.%) coating as 0.084 × 10^−6^ Ω·m, 0.120 × 10^−6^ Ω·m, 0.128×10^−6^ Ω·m, 0.144 × 10^−6^ Ω·m, and 0.173 × 10^−6^ Ω·m. Compared to other copper alloy coating systems [4,5,6,7,8,9,10] (with a hardness of 684 HV and a resistivity of 3.51 × 10^−6^ Ω·m), the coating still maintains a relatively high electrical conductivity. From the conductive mechanism of metals [33], when an electron wave passes through an ideal crystal lattice at absolute zero, it will propagate unhindered by scattering, and the material is an ideal conductor. In fact, there are reinforcement particles, dislocations, and solve-solution atoms in the Cu-MoSi_2_ coating system (Figure 13a), all of which disrupt the periodicity of the crystal lattice to varying degrees. According to Matthiessen’s Rule [34]:

(1) $$\rho =\rho (T)+\rho^{\prime }$$

The total resistance of the metal (ρ) includes the basic resistance of the metal (ρ(T)) and the impurity resistance ($\rho^{\prime }$); (ρ(T)) is temperature-dependent; ($\rho^{\prime }$) is related to the impurity concentration and defect. Among them, Si atoms in solid solution have the greatest influence on resistivity. In this regard, the first principles are used to analyze the effect of Si atoms in Cu-Si solid solution on the conductivity. In order to simulate the solid solution of Si atoms in copper crystals, we established a 2 × 2 × 2 supercell model (a total of 32 copper atoms) and dissolved different amounts of Si atoms into the supercell to obtain Cu-Si solid solutions with solid solubility of 0%, 3.03%, and 5.88%, respectively. The electrical conductivity of the solid solution is measured by the average number of free electrons. As shown in Figure 13b, the average number of free electrons of Cu-0Si, Cu-3.03Si, and Cu-5.88Si is 10.9977, 10.7864, and 10.5870, respectively. The average number of free electrons decreases with the increase in solid solubility, which means that the conductivity decreases. This is mainly caused by two aspects. From the perspective of the crystal structure of Cu-Si solid solution, the solidly dissolved Si atoms lead to lattice distortion and destroy the periodicity of the lattice arrangement of copper crystals. From the difference in charge density, the Cu atom and Si atom have obvious electron transfer, and the chemical bond generated is strong, resulting in the reduction of valence electrons. Different from other work using in situ generation of Mo and Si atoms to introduce the MoSi_2_ enhancement phase [35], we directly added MoSi_2_ particles, which greatly reduced the solid solution concentration of Si atoms, which is an important reason why Cu-MoSi_2_ coating can maintain high hardness while maintaining electrical conductivity. In addition, the high-speed laser cladding technology has the characteristics of rapid solidification [36]. This method shortens the diffusion time of Si atoms decomposed by MoSi_2_, which is conducive to the preparation of Cu-MoSi_2_ coatings with high hardness, wear resistance, and good electrical conductivity.

## 4. Conclusions

Utilizing high-speed laser cladding technology, a novel Cu-MoSi_2_ coating with exceptional properties of super hardness, wear resistance, and high electrical conductivity was successfully prepared on the surface of CuCrZr alloy. The principal conclusions are as follows:

1. A Cu-MoSi_2_ coating was fabricated on the surface of copper alloy substrate, with enhanced particle dispersion of MoSi_2_. Notably, the Cu-30%MoSi_2_ coating exhibited the highest hardness (974.5 HV_0.5_) and the lowest wear amount (0.0619 mg/km). This corresponds to an approximately 15-fold increase in hardness compared to the copper base material (65 HV_0.5_), and a mere 0.45% of the wear amount (13.71 mg/km). Furthermore, the coating demonstrated a resistivity of 0.173 × 10^−6^ Ω·m, effectively fulfilling the requirements for both high hardness, wear resistance, and electrical conductivity.
2. The elevated hardness and wear resistance performance were intricately intertwined with the dispersion of reinforcing Mo_x_Si_y_ particles. With the augmentation of MoSi_2_ content, microstructural refinement occurred, accompanied by a gradual and uniform dispersion of Mo_x_Si_y_ reinforcing particles. This orchestrated enhancement significantly elevated the coating’s hardness, concurrently altering the frictional wear mechanism and markedly ameliorating wear resistance.
3. The adoption of direct incorporation of MoSi_2_ particles emerged as a pivotal factor enabling the preservation of favorable electrical conductivity within the coating. Employing first-principle analysis, it is ascertained that solid solution Si atoms constitute the principal culprits contributing to conductivity reduction. By adopting the strategy of direct MoSi_2_ particle incorporation, the extent of Si atom solid solution was effectively circumvented. As a consequence, despite a modest increase in resistivity, the coating’s electrical conductivity exhibited nominal variance, thereby substantiating the judiciousness of the chosen approach.

In summation, the present study underscores the achievement of an innovative Cu-MoSi_2_ coating via high-speed laser cladding technology, thereby manifesting a triumvirate of attributes encompassing heightened hardness, wear resistance, and elevated electrical conductivity. This multifaceted achievement holds promising implications for diverse applications requiring a confluence of these salient attributes.

## Figures and Tables

**Table 1:** Powder composition for Cu-MoSi_2_ composite coatings (wt.%).

Compositions	Cu (wt.%)	MoSi_2_ (wt.%)
Cu-10MoSi_2_	90	10
Cu-15MoSi_2_	85	15
Cu-20MoSi_2_	80	20
Cu-25MoSi_2_	75	25
Cu-30MoSi_2_	70	30

## Data Availability

Data are contained within the article.
